# Mucopolysaccharidosis VI

**DOI:** 10.1186/1750-1172-5-5

**Published:** 2010-04-12

**Authors:** Vassili Valayannopoulos, Helen Nicely, Paul Harmatz, Sean Turbeville

**Affiliations:** 1 Reference Center for Inherited Metabolic Diseases, Necker-Enfants Malades Hospital, Paris, France; 2 BioMarin Pharmaceutical Inc., Novato, California, USA; 3 Children's Hospital & Research Center Oakland, Oakland, California, USA

## Abstract

Mucopolysaccharidosis VI (MPS VI) is a lysosomal storage disease with progressive multisystem involvement, associated with a deficiency of arylsulfatase B leading to the accumulation of dermatan sulfate. Birth prevalence is between 1 in 43,261 and 1 in 1,505,160 live births. The disorder shows a wide spectrum of symptoms from slowly to rapidly progressing forms. The characteristic skeletal dysplasia includes short stature, dysostosis multiplex and degenerative joint disease. Rapidly progressing forms may have onset from birth, elevated urinary glycosaminoglycans (generally >100 μg/mg creatinine), severe dysostosis multiplex, short stature, and death before the 2nd or 3rd decades. A more slowly progressing form has been described as having later onset, mildly elevated glycosaminoglycans (generally <100 μg/mg creatinine), mild dysostosis multiplex, with death in the 4th or 5th decades. Other clinical findings may include cardiac valve disease, reduced pulmonary function, hepatosplenomegaly, sinusitis, otitis media, hearing loss, sleep apnea, corneal clouding, carpal tunnel disease, and inguinal or umbilical hernia. Although intellectual deficit is generally absent in MPS VI, central nervous system findings may include cervical cord compression caused by cervical spinal instability, meningeal thickening and/or bony stenosis, communicating hydrocephalus, optic nerve atrophy and blindness. The disorder is transmitted in an autosomal recessive manner and is caused by mutations in the *ARSB *gene, located in chromosome 5 (5q13-5q14). Over 130 *ARSB *mutations have been reported, causing absent or reduced arylsulfatase B (*N*-acetylgalactosamine 4-sulfatase) activity and interrupted dermatan sulfate and chondroitin sulfate degradation. Diagnosis generally requires evidence of clinical phenotype, arylsulfatase B enzyme activity <10% of the lower limit of normal in cultured fibroblasts or isolated leukocytes, and demonstration of a normal activity of a different sulfatase enzyme (to exclude multiple sulfatase deficiency). The finding of elevated urinary dermatan sulfate with the absence of heparan sulfate is supportive. In addition to multiple sulfatase deficiency, the differential diagnosis should also include other forms of MPS (MPS I, II IVA, VII), sialidosis and mucolipidosis. Before enzyme replacement therapy (ERT) with galsulfase (Naglazyme^®^), clinical management was limited to supportive care and hematopoietic stem cell transplantation. Galsulfase is now widely available and is a specific therapy providing improved endurance with an acceptable safety profile. Prognosis is variable depending on the age of onset, rate of disease progression, age at initiation of ERT and on the quality of the medical care provided.

## Disease name with synonyms

Mucopolysaccharidosis VI; MPS VI; MPS 6

Maroteaux-Lamy syndrome

*N-*acetylgalactosamine 4-sulfatase deficiency

Arylsulfatase B deficiency; ASB deficiency

## Definition

Mucopolysaccharidosis VI (MPS VI) or Maroteaux-Lamy syndrome (MIM # 253200) is an autosomal recessive lysosomal storage disorder described in 1963 by Dr. Pierre Maroteaux and Dr. Maurice Lamy [1] and determined by mutations in the arylsulfatase B (*ARSB*) gene located in chromosome 5 (5q13-5q14)[2]. Pathogenic mutations of this gene result in reduced or absent activity of the enzyme arylsulfatase B (ASB) also called *N*-acetylgalactosamine 4-sulfatase (E.C.3.1.6.12) leading to incomplete degradation and cellular accumulation of the glycosaminoglycan(s) (GAG), (previously also known as a "mucopolysaccharide" dermatan sulfate (DS)), and cell injury. Another GAG, chondroitin 4-sulfate (CS), is also a substrate for ASB [3,4] but is hydrolyzed by hyaluronidase and β-glucuronidase to trisaccharides and higher oligosaccharides that also accumulate but are not recognized as "classic storage material" [J Hopwood, personal communication]. Clinical manifestations are related to progressive accumulation of DS GAG and sulfated oligosaccharides derived from both DS and CS in lysosomes, cells and tissues.

## Epidemiology

The epidemiological studies of MPS VI are limited to publications describing birth prevalence whereas no studies describing population prevalence are available. These birth prevalence studies are based on clinical identification of patients and regional birth rate [5] as represented in Table 1. They range from 1 in 43,261 births in Turkish immigrants living in Germany [6] to 1 in 1,505,160 births in Sweden [7]. Because these birth prevalence estimates are derived from patient referrals based mostly on clinical identification, (except for a single publication that mentions prenatal diagnosis for one patient in Australia [8]), they will be underestimates of the true birth prevalence, which will only be determined when newborn screening becomes available. Because of the limited number of countries involved in these estimates, it is not possible to generate a worldwide estimate of birth prevalence. In the US, the absence of centralized laboratory diagnosis makes it difficult to provide birth prevalence for MPS VI as was completed in the above surveys.

**Table 1:** Estimated Birth Prevalence of MPS VI.

Reference	Country (sub-population data within citations in parentheses)	Study Years	Number of diagnosed patients with MPS VI	Prevalence per 10^5^ live births	Incidence Rate Per Total Live Births	MPS VI as % of total MPS diseases for each country.
Meikle et al 1999[8]	Australia	1980-1996	18**	0.43	1 in 248,372	10.36%

Nelson et al 2003[117]	Western Australia	1969-1996	2	0.31	1 in 320,589	*

Coelho et al 1997[11]	Brazil	1982-1995	39	*	*	18.48%

Applegarth et al 2000[118]	British Columbia, Canada	1972-1996	1	0.1	1 in 1,035,816	5.0%

Lowry et al 1990[119]***	British Columbia, Canada	1962-1986	1	*	1 in 936,572	5.7%

Baehner et al 2005[6]	Germany (Turks in Germany)	1980-1995	31 (16)	0.23 (2.3)	1 in 432,610 (1 in 43,261)	6.96% (4.42%)

Michelakakis et al 1995[120]	Greece	1982-1994	3	*	*	*

Poorthuis et al 1999[9]	Netherlands	1970-1990	6	0.15	*	3.30%

Nelson et al 1997[121]	Northern Ireland	1958-1985	0	0	Zero cases in 840,000	0

Pinto et al 2004[10]	Portugal (Northern Portugal)	1982-1999 1962-1999	16 (10)	* (0.42)	* (1 in 238,095)	* (16.12%)

Lin et al 2008[122]	Taiwan	2000-2006	2	*	1 in 833,000	*

Malm et al 2008[7]	Sweden	1975-2004	2	0.07	1 in 1,505,160	4%
	Norway	1979-2004	1	0.07	1 in 1,455,813	2%
	Denmark	1975-2004	2	0.05	*	3%

*Data not available.

**Prenatal and postnatal data.

***Lowry et al, 1990[119] reported one case over a 44-year period from 1952-1986, although this period includes a period prior to the first description of MPS VI in 1963. It is unclear if this is the same case reported by Applegarth et al, 2000[118], for the same population. Also the period given in Table 1 by Lowry et al, 1990[119], is corrected from the previously cited birth prevalence in MPS VI literature to represent here the live births reported in the more accurate period from 1962 to 1986 as this is the closest period in the report to the first description of MPS VI in 1963.

The relative frequency of MPS VI compared to other mucopolysaccharidoses (MPS) was reported where possible. As shown in Table 1, MPS VI ranges from 2-4% of all MPS in Scandinavia (Sweden, Norway and Denmark) [7] and 3% in the Netherlands [9], to highs of 16% in Portugal [10] and 18.5% in Brazil [11].

Although no specific ethnic group has been associated with an increased risk of MPS VI, some populations have been found to have increased frequencies of specific mutations. As described by Petry et al 2003 and 2005 [12,13], there is a common mutation 1533del23 among Brazilian MPS VI patients found in 23% of alleles, which also occurs in Portuguese MPS VI patients, although the frequency is unknown.

Very high birth prevalence was identified in the Turkish population living in Germany in contrast to the non-Turkish German population (1 in 43,261 vs. 1 in 432,610, respectively) [6]. Baehner et al, (2005) suggest that this might be related to a high degree of consanguinity within this ethnic population. Since birth prevalence appears to be 10-fold higher among Turkish Germans than non-Turkish Germans, we may be greatly underestimating MPS VI birth prevalence in selected regions or world-wide if it is based on earlier country surveys with populations having a low frequency of consanguinity.

## Clinical description

The clinical presentation of MPS VI varies greatly with respect to age of onset and rate of disease progression. Swiedler et al, (2005) [14] conducted a cross-sectional survey of a group of 121 untreated MPS VI patients and suggested that two broad classification groups could be characterized based on height and urinary GAG (uGAG) levels. High uGAG levels (>200 μg/mg creatinine) were associated with an advanced clinical course relative to age characterized by short stature (height range 80 cm to 120 cm), low body weight, impaired endurance based on a walk test, compromised pulmonary function, and reduced joint range of motion. Age greater than 20 years was rarely found with uGAG levels above a threshold of 100 μg/mg creatinine, suggesting uGAG < 100 μg/mg creatinine might predict longer survival. Those with slowly progressing disease tended to have heights above 140 cm and uGAG levels below 100 μg/mg creatinine.

Although descriptive classification systems have mostly described patients as rapidly progressing (with severe symptoms) or slowly progressing (with mild or attenuated symptoms), an intermediate stage has also been described [14-20]. Despite the utility of communicating with these descriptive categories, it is important to recognize that the disease manifests symptoms along a continuum. Compared to the rapidly progressing disease where severe symptoms occur in several systems simultaneously, the slowly progressing disease may have clinically significant symptoms occurring in fewer systems[17]. For example, severe symptoms may progress in a single organ system within the slowly progressing teenage or adult patient to an extent of requiring surgery (e.g., hip replacement, cardiac valve replacement, corneal transplantation, or cervical spinal cord decompression[16]). In a recent publication, Karageorgos et al 2007 [15] have classified certain gene mutations of the *ARSB *gene as likely to result in phenotypes falling into the descriptive categories of rapidly progressing, intermediate, and slowly progressing disease. Despite the authors' separation of gene mutations into three groups by severity among MPS VI patients, it is important to emphasize that there are no fixed parameters for separating these descriptive categories. The 105 patients studied by Karageorgos et al (2007) were participants in the BioMarin Phase 1/2, Phase 2 or Survey Study, and phenotype correlations were generated from clinical data described in these studies.

### Rapidly Progressing MPS VI

**Figure 1:**
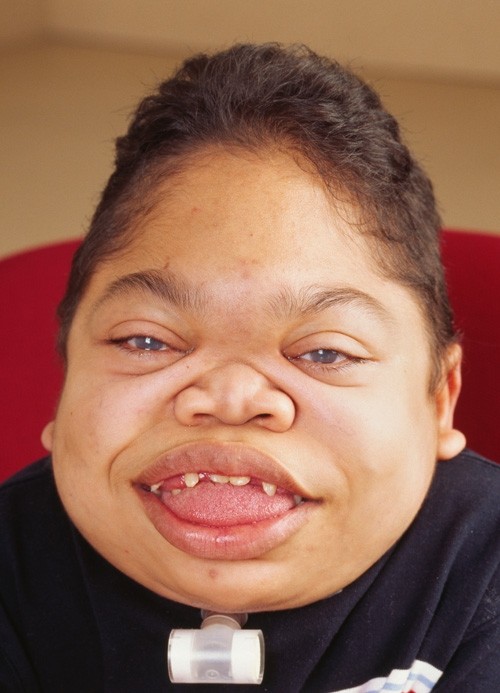
**Rapidly progressing 16-year old MPS VI patient: Photograph of face showing coarse facies: frontal bossing, enlarged tongue, thick lips, abnormal dentition and gingival hyperplasia**.

**Figure 2:**
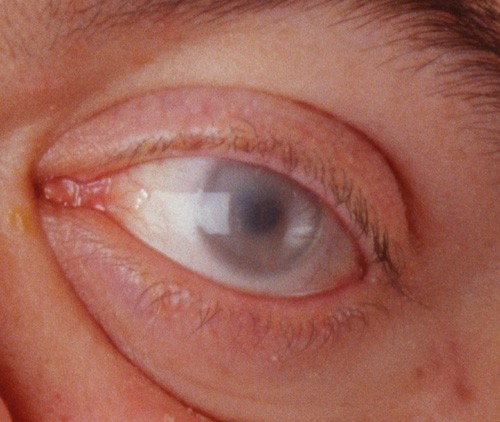
**Rapidly progressing 30-year old male MPS VI patient: Photograph of eye with corneal clouding**.

**Figure 3:**
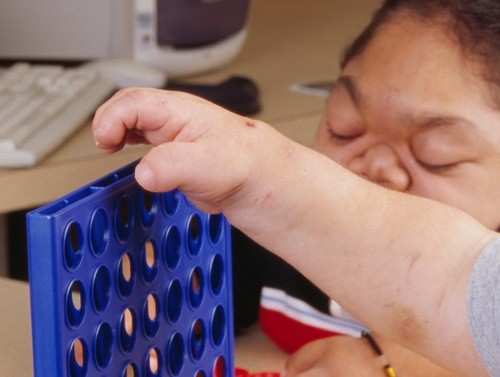
**Rapidly progressing 16-year old male MPS VI patient: Photograph of claw-hand deformity**.

**Figure 4:**
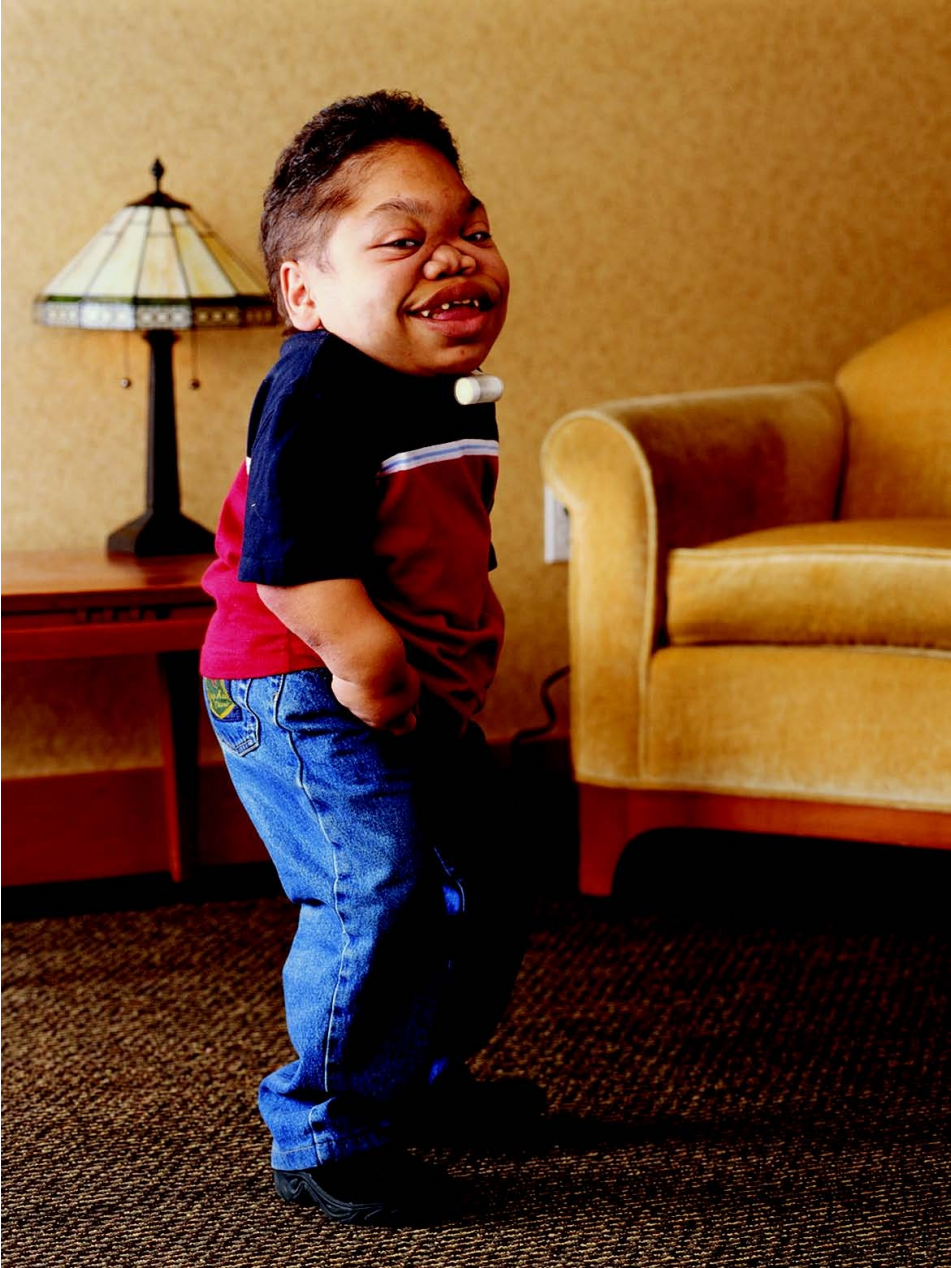
**Rapidly progressing 16-year old male MPS VI patient: Photograph of patient showing curvature of spine (lumbar kyphosis, scoliosis, lordosis)**.

The rapidly progressing form has correlation to certain alleles described in the literature [15], is characterized in most cases by onset before 2 or 3 years of age, impaired mobility by 10 years of age, absent or delayed puberty, cervical spinal cord compression, respiratory insufficiency and surgical complications. Patients with the rapidly progressing form were frequently reported to die from heart failure in the 2nd or 3rd decades [21]. From the cross-sectional survey [14], one can deduce that the higher the uGAG, the faster is the rate of clinical development. Growth often slows after the first year of life with complete cessation at 3 to 4 years of age. Adult height is generally less than 120 cm [14]. Other physical findings may include thoracic deformity (pectus carinatum), stiff and contracted joints, scoliosis or kyphosis (gibbus malformation), macrocephaly, hepatosplenomegaly, protruding abdomen, umbilical and/or inguinal hernia, coarse facial features (Fig 1) including frontal bossing, a depressed nasal bridge, enlarged tongue, gingival hypertrophy, delayed dental eruption, and hypertrichosis (hirsutism). Patients may have labored breathing, loud snoring with sleep apnea, thick nasal discharge, frequent sinusitis or otitis media arising from narrowed airways and thick mucous secretions [22]. Hearing loss, involving both conductive and neurosensory mechanisms, is common and may lead to the impression that the patient may have developmental delay. Vision is often compromised by slowly increasing corneal clouding (Fig 2), but can also show rapid deterioration related to optic nerve damage from increased intracranial pressure or compression along the optic nerve [23]. Patients older than 10 years may progress to severe pulmonary obstruction and respiratory failure requiring tracheostomy [24], cardiac valve regurgitation or stenosis requiring valve replacement, severe joint disease [25] especially of the hips that may require replacement, claw-hand deformities (Fig 3) secondary to flexion contractures and carpal tunnel disease requiring median nerve release, severe spinal kyphosis, scoliosis (Fig 4), and cervical stenosis with spinal cord compression [26,21] requiring decompression and possible stabilization to prevent paralysis. These severe symptoms occurring together are typical of rapidly progressing disease and result in multiple hospital visits and surgical procedures accompanied by high-risk anesthesia [26]. In general, patients of the rapidly progressing disease type have uGAG elevated above those levels observed in slowly progressing patients.

### Slowly Progressing MPS VI

**Figure 5:**
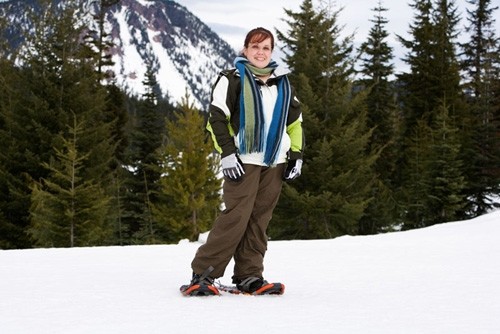
**Slowly progressing female MPS VI patient (aged in her late twenties)**.

**Figure 6:**
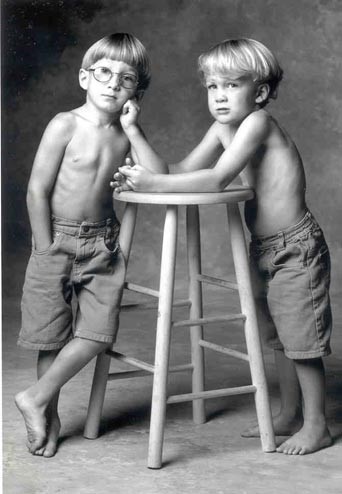
**Slowly progressing MPS VI male patients aged 7 years old (right) and aged 8 years old (left): Patients received BMT but both failed enduring engraftment of BMT and continued on ERT**.

The slowly progressing disease form (Fig 5 and Fig 6) is characterized by later onset of symptoms due to lower levels of dermatan sulfate [14]. Symptoms may not at first appear in a recognized pattern, but when apparent, usually in teen years or early adulthood, the milder symptoms of MPS disease may be observed by an alert physician and lead ultimately to diagnosis [21,27,28]. Despite classification as slowly progressive, these patients may develop skeletal complications including carpal tunnel disease, hip disease and a decrease in their overall functional status by the late teenage years [14]. Diagnosis generally occurs after 5 years of age and may be delayed until the 2^nd ^or 3^rd ^decades. However, most patients with MPS VI will develop serious manifestations of the disease at some point including joint degeneration, cardiac valve disease, sleep apnea, a decrease in pulmonary function and reduced endurance [28]. Evidence of a therapeutic response following treatment with galsulfase in five slowly progressing adult patients was reported recently by Lampe et al 2008 [27].

A case history presented by Brooks et al, (2005) [29] describes a very mild case of a female, aged 44 years, at the extreme end of the scale of the slowly progressing patients, without visible or clinical symptoms of MPS VI, who was detected upon routine blood examination to have inclusion bodies in lysosomes of white blood cells, suggestive of a lysosomal storage disorder (LSD). She had a 5% level of the normal ASB catalytic activity, slight dermatan sulfaturia, halitosis, slight photophobia, dyslexia, mild hearing loss, normal height and mild hepatomegaly (1 cm) without splenomegaly. Patients presenting with such mild characteristics are not easily recognized as having MPS VI disease until a pattern of findings has been well established, such as cardiac valve abnormalities, corneal clouding, reduced endurance and repeated respiratory and sinus infections atypical for a normal age-matched group, and spine, hip or hand skeletal disease. The pathophysiology is similarly related to progressive accumulation of GAG that affects structure and function of involved organs but is due to a lower concentration of primary storage material than in rapidly progressing disease, and consequently leads to a slower rate of clinical progression.

### Skeletal Disease

**Figure 7:**
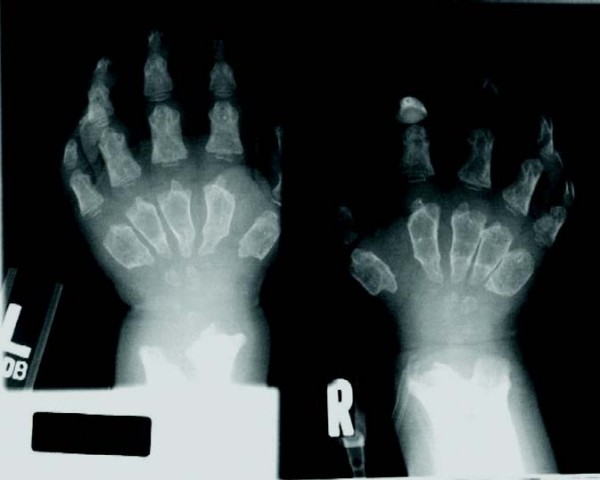
**Rapidly progressing 3-year old male MPS VI patient: radiograph of hand**.

**Figure 8:**
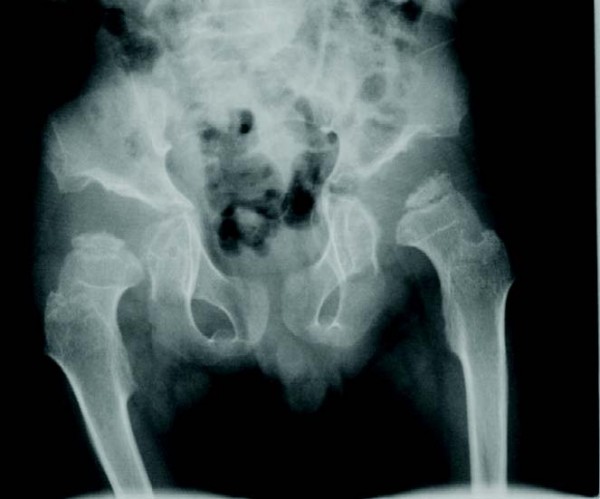
**Rapidly progressing 9-year old female MPS VI patient: radiograph of hip showing dysplastic femoral head and severe hip dysplasia**.

**Figure 9:**
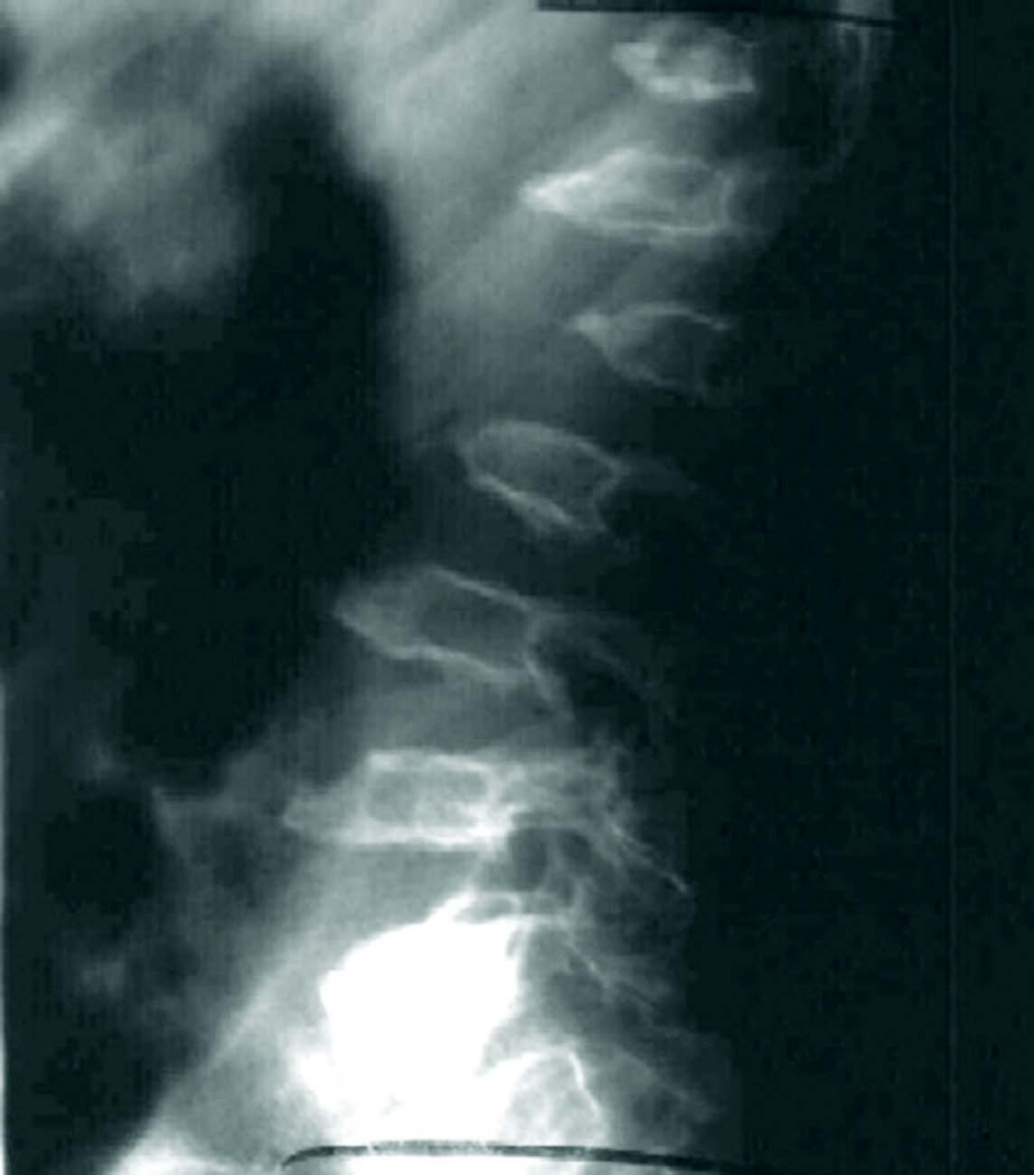
**Rapidly progressing 8-year old male MPS VI patient: radiograph of spinal column showing abnormal development of vertebral bodies, paddle-shaped widened ribs and short, thick irregular clavicles, lordosis, kyphosis and scoliosis**.

**Figure 10:**
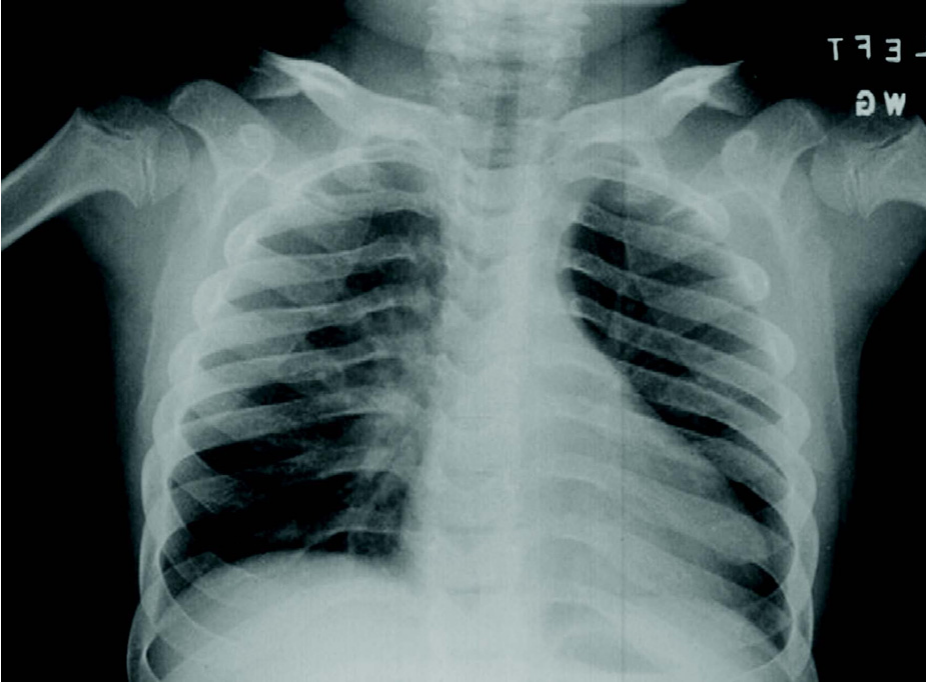
**Rapidly progressing 12-year old male MPS VI patient: radiograph of clavicles and thorax**.

**Figure 11:**
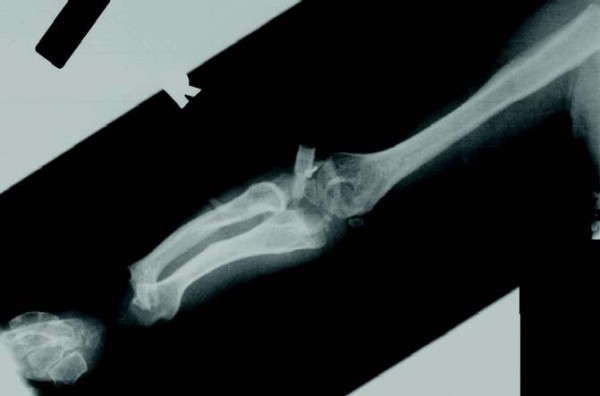
**Rapidly progressing 9-year old female MPS VI patient: radiograph of arm showing ulna radius and humerus**.

**Figure 12:**
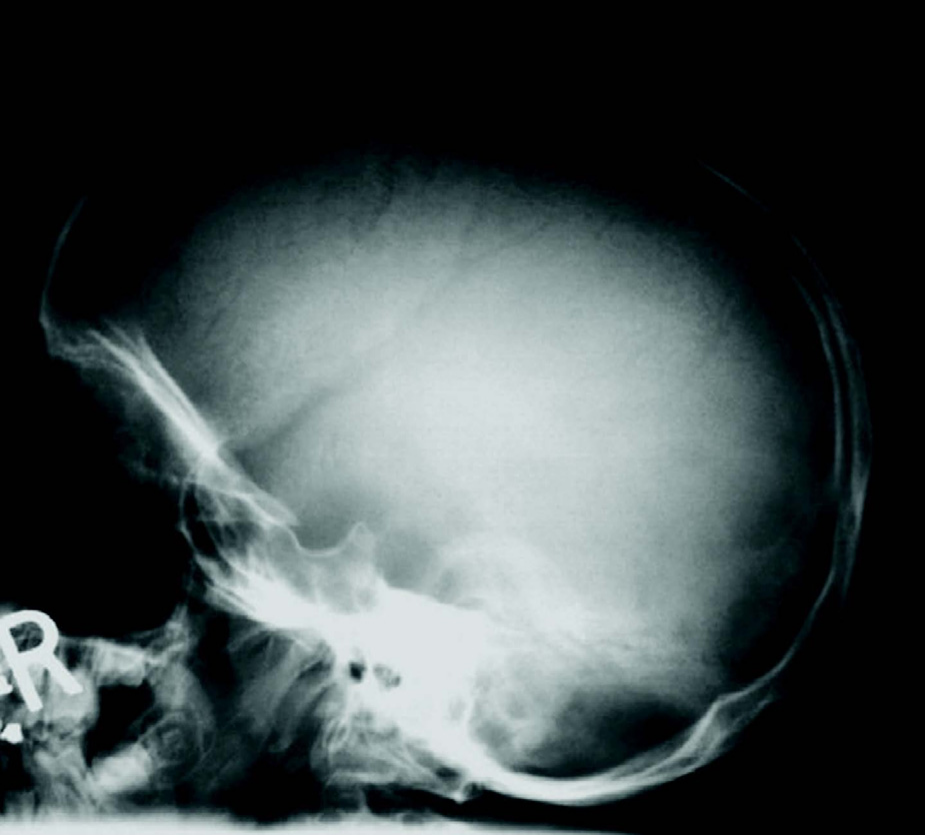
**Rapidly progressing 5-year old female MPS VI patient: radiograph of cranium showing thickened diploic space and J-shaped sella**.

The radiological findings that are characteristic of MPS VI, other MPS, mucolipidoses (ML II I-Cell disease/Leroy, ML III (pseudo-Hurler polydystrophy) and other storage diseases (multiple sulfatase deficiency, carbohydrate-deficient glycoprotein syndrome, GM I gangliosidosis and geleophysic dysplasia) are given the term "dysostosis multiplex". Typical radiological findings include thickened, short metacarpal bones (Fig 7) with proximal pointing and thin cortices, carpal bones that are irregular and hypoplastic and tarsal bones that have irregular contours, a dysplastic femoral head, severe hip dysplasia (Fig 8), abnormal development of vertebral bodies of the spine (Fig 9), paddle-shaped widened ribs and short, thick irregular clavicles (Fig 10), hypoplastic distal ulna and radius (Fig 11), thickened diploic space and abnormally shaped J-shaped sella in the cranium (Fig 12). Slowly progressing MPS VI patients may not demonstrate all the above characteristics of dysostosis multiplex.

### Ear, Nose and Throat (ENT)-Related Disease

ENT disease in MPS VI often involves hearing disorders, otitis media, as well as oral, pharyngeal, and upper airway obstruction. The general pathophysiology of ENT disease in MPS is related to GAG deposition in mucosae and in other tissue layers such as those of tongue and tonsils, with progressive airway narrowing of the nasopharynx and oropharynx[30]. Increased production of mucous secretions leads to rhinorrhea, sinus infections, and otitis media by occluding sinus drainage and blockage of the Eustachian tubes. Permanent hearing loss is common and believed to be conductive and neurosensory in nature. The physical bulk of tissues distended by GAG storage product in the posterior pharynx with possible prolapse of excessive tissue into the larynx may cause stridor and compromise airways[22].

Upper airway obstruction leading to obstructive sleep apnea is a common morbidity; sleep apnea is assessed using polysomnography.

Special precautions with general anesthesia and surgery surround the short stiff neck, large amount of obstructing oral pharyngeal and upper airway tissue, and risk of spinal cord injury if neck is hyperextended. It is recommended that tonsillectomy, adenoidectomy and other head and neck surgical procedures be performed without extending the neck. For the same reason, normal intubation for anesthesia is not usually possible and should only be performed by anesthesiologists skilled in flexible bronchoscopy-assisted intubation and who are aware of the severe difficulties that are present in these patients [31-33].

### Pulmonary Disease

Patients with MPS VI may have features of obstructive and restrictive lung disease. The obstructive lung disease is related to narrowed bronchial airways [30] and tracheobronchomalacia, a weakness that can lead to acute airway obstruction or collapse, whereas the restrictive lung disease is due to a small, stiff thoracic cage, and abdominal distention, combined with kyphosis, scoliosis, and increased lumbar lordosis[34].

Disease complications include recurring episodes of pneumonia. Evaluation of pulmonary function by spirometry including flow-volume expiratory and inspiratory loops should be performed regularly to assess changes in lung volume and obstruction. Reference values for the normal population may not be appropriate for use in evaluating MPS VI pulmonary function tests. However, it is useful to follow absolute respiratory volume parameters longitudinally. Fiber-optic bronchoscopy can also be performed to assess severity of airway obstruction and tracheomalacia. It is particularly helpful to assess the airway before anesthesia to assist in planning for this procedure. Vaccinations against respiratory pathogens causing influenza and pneumococcus infections should be considered to prevent pneumonia from these organisms.

### Cardiac Disease

Cardiac abnormalities are frequent in patients with MPS VI and are an important cause of morbidity and mortality [14,35-37]. Heart disease has been shown using serial echocardiography to progressively worsen with age in even the slowly progressive patients[37]. Patients develop stenosis and/or insufficiency as demonstrated in a study of 28 MPS VI patients [36] with disease in the mitral valves (96%), tricuspid valves (71%) and aortic valves (43%). The literature pertaining to MPS VI reveals cardiomyopathy and cardiac failure in a 5-month-old infant with MPS VI [38] and endocarditis fibroelastosis and cardiac failure in a 9-month-old with MPS VI [39]. Cardiac evaluations are recommended every 1 to 2 years and should include obtaining a blood pressure reading, performing electrocardiography and echocardiography to assess abnormal cardiac rhythm or conduction abnormality or changes in heart structure or function.

Endocarditis is also a consideration in MPS VI patients fitted with a mediport (central venous access port) for infusion of ERT. No literature yet supports infection risk for MPS VI but the literature for hemophiliacs suggests that central venous ports have a risk of infection at a rate of 0.45 per 1000 catheter days. Replacement of aortic and mitral valves, singly or in combination, has been performed for MPS VI [40] and should be considered for severe valve stenosis or regurgitation.

Systemic hypertension is common in approximately 20% of MPS I patients and may be considered in those patients with MPS VI [26]. Hypertension may develop in relation to aortic or renal artery narrowing or chronic intermittent hypoxia. The need for antibiotics to prevent subacute bacterial endocarditis and valvular disease from surgical interventions and dental procedures should be discussed with a cardiologist. Although not reported for MPS VI, there may be a risk of coronary artery disease based on studies in MPS I patients that suggest additional care is required during surgery to avoid hypotension and cardiac ischemia.

### Ophthalmological Disease

Ashworth et al (2006) [41] have published a review of the ophthalmology of the MPS and note that MPS VI patients were particularly prone to glaucoma (50%) and corneal clouding (95%), which may be treated with medication and corneal transplantation surgery, respectively. Thickening of the cornea can result in spurious readings of high intraocular pressure (IOP) and prevent visualization of the retina. Increased IOP may be related to narrowing of the anterior chamber angle by iridociliary cysts (closed angle glaucoma) or GAG deposition in the trabecular cells blocking reabsorption (open-angle glaucoma). Corneal transplantation may lead to normalization of spurious IOP measurements.

Optic nerve abnormalities may be due to GAG accumulation in optic nerve ganglion cells, compression of the optic nerve by thickened dura or bony narrowing along the optic nerve tract, or increased intracranial pressure (ICP). Fifty percent of MPS VI patients have an abnormal optic disk with mild to moderate swelling; 15% have optic nerve atrophy. Retinopathy is rare but has been reported [41,42] and night blindness or dimness may be a reported symptom.

MPS VI patients should be evaluated annually for strabismus, visual acuity, refraction and IOP by tonometry if possible. A fundoscopic examination should be performed after dilation to evaluate the retina and optic nerve; and photographs taken if possible. Visual fields should be assessed when there are changes in optic nerve appearance, or if increased IOP is recorded. Visual evoked-potential examinations may be used to evaluate the function or health of the optic nerve while severe corneal clouding prevents fundoscopic examination of the optic disk.

Interventions include corrective lenses, medications, and/or surgery to control increased IOP, patching for amblyopia, or surgery to correct strabismus when relevant. In addition, corneal transplantation (penetrating keratoplasty) may be performed to correct severe corneal clouding with vision loss. Retinopathy is rare but has been reported [41,42] and night blindness or dimness may be a reported symptom.

### Central Nervous System Disease

Most MPS VI patients have relatively normal intellectual development [43], unlike MPS I Hurler, or severe MPS II Hunter syndrome [21]. Although severe intellectual deficit has been reported in two families with arylsulfatase B deficiency [44,45], it is not clear that this was caused by the MPS disease *per se *[21]. Valayannopoulos et al 2009, reported an unusually high prevalence of intellectual deficit occurring in 4 of 6 French MPS VI patients followed at one center[46].

Vedolin et al, (2007) [47] report a study involving 17 MPS VI patients and mentioned a higher cerebral volume in MPS VI patients compared to other MPS type patients, and described extensive white matter lesions and ventricular enlargement in some (exact number undisclosed) of the 17 MPS VI patients. It is not known why MPS VI patients are spared deficits in cognitive neurological development [48] although absence of heparan sulfate (a storage substrate in MPS I, II, III and VII) GAG storage is probably important.

The frequent vision loss combined with a hearing impairment due to a combination of conductive and neurosensory loss may contribute to or be misinterpreted as intellectual impairment in MPS VI patients.

MPS VI patients may also develop communicating hydrocephalus and increased ICP [49] due to GAG occlusion of the subarachnoid reabsorptive apparatus. Typical symptoms of communicating hydrocephalus such as morning headaches and vomiting are often absent, although some patients may present with rapid visual deterioration. The diagnosis of increased ICP may be suggested by computed tomography or MRI although ventricular dilation may also be related to cortical atrophy. Direct measurement of central nervous system pressure may be required for diagnosis. If increased ICP is documented, a ventriculoperitoneal shunt may prevent optic atrophy and vision loss in some patients. This rapid onset of blindness may also be caused by compression occurring along the optic nerve, and decompression of this nerve may also be necessary[50].

Patients can also develop cervical spinal cord compression and injury from bony abnormalities and dural thickening. Spinal cord decompression surgery can help reduce spinal cord compression and prevent loss of function. A study of intrathecal delivery of ERT has not yet been initiated in the setting of MPS VI, although a similar route of ERT administration was conducted in an MPS I patient[51].

## Etiopathogenesis

MPS VI is an autosomal recessive disease resulting from mutations in the chromosomal position 5q13-5q14 [2]. Most of the 133 mutations reported by the Human Gene Mutation Database in June, 2009, are missense or non-sense mutations (n = 100), and in addition 9 splicing mutations, 18 small deletions, 3 small insertions, 1 small "indel"(insertion and deletion), and 2 gross deletions have also been documented. One of these 133 mutations (p.T212I) has pseudo-deficient enzyme activity, a normal phenotype and normal uGAG levels [52].

Disease manifestations are present only in patients with severe deficiency in enzymatic activity, which is usually below 10% of the lower limit of normal[29]. The greatest severity of symptoms is found in patients who have no detectable enzyme activity. Carriers with only one abnormal allele (the other allele being normal) do not exhibit any clinical findings due to sufficient activity of ASB enzyme.

Each disease-causing mutation impacts the production of a functional ASB enzyme that catalyzes the cleavage of the sulfate ester from non-reducing terminal *N *-acetylgalactosamine 4-sulfate residues, thereby affecting the catabolism of sulfated oligosaccharide substrates [3]: CS and DS. Lengths of DS polysaccharides and oligosaccharides are determined by the relative amount of iduronic acid relative to glucuronic acid present in the DS chain. CS is digested by hyaluronidase to sulfated tetrasaccharides that are further reduced by the action of β-glucuronidase to sulfated trisaccharides with non-reducing end *N*-acetylgalactosamine 4-sulfate residues that accumulate as undigested substrate (J Hopwood, personal communication). The glucuronic acid residues enable the hyaluronidase digestion of the DS chains to oligosaccharides with non-reducing end β-linked glucuronic acid residues that may be removed by the action of β-glucuronidase. DS, also known as chondroitin sulfate B [53], is a necessary component in the formation of structural proteins that form connective tissue, such as collagen and bone.

The rapidly progressing feline MPS VI model (used to demonstrate efficacy of enzyme replacement therapy [54,55]), is due to the homozygous presence of p.L476P mutation and expresses approximately 0.3% of normal white blood cell ASB activities [56]. This severe phenotype includes dwarfism, facial dysmorphia, degenerative joint disease, corneal clouding, lysosomal inclusions in most tissues including chondrocytes and abnormal leukocyte inclusions. The compound heterozygous presence of a mild mutation p.D520N with p.L476P enables the expression of approximately 1% of normal white blood cell ASB activity. The phenotype of the compound heterozygote includes clear corneas, normal connective tissue inclusions, mild inclusions in chondrocytes, mild joint disease and normal skeletal growth [57]. This double heterozygote identifies the limit of ASB activity needed to prevent MPS VI skeletal disease in the cat as approximately 1% of normal white blood cell ASB. Homozygous presentation of the p.D520N mutation, associated with the expression of approximately 2% of normal white blood cell activity, leads to disease-free cats although inclusion bodies can be identified in neutrophils. Thus in cats, the range of 0.3% to 2% of normal ASB activity in white blood cells covers the range of clinical severity of the MPS VI phenotype [57].

Accumulation of GAG DS causes lysosomal engorgement, cell enlargement and dysfunction in animal models of MPS VI, as is found in human MPS VI [21]. The accumulation of DS specifically is the primary pathological defect. Secondary pathological processes also play an important role in pathophysiology. GAG has been shown to stimulate inflammatory response in articular chondrocytes through lipopolysaccharide (LPS)-mediated signaling [58-60] in animal models of MPS disease. GAG has been shown to play a similar role in other connective tissue diseases [61].

### Problems with Genotype-Phenotype Correlations

It has been difficult to determine genotype-phenotype correlation for most patients. This results primarily because a large number of mutations have been identified and in most cases patients have either a novel or private mutation or share a mutation with one or two individuals and clear descriptions of the phenotype were not provided. As an autosomal recessive disease, MPS VI requires the presence of two mutated copies of the *ARSB *gene, located on chromosome 5 for disease expression. If a patient has the same *ARSB *mutation on each one of his chromosome 5, the patient is homozygous and there exists the best chance of making a genotype-phenotype correlation. If the patient has two different *ARSB *mutations, one on each chromosome 5, the patient is compound heterozygous. In this case, it is more difficult to determine the phenotype. In addition a polymorphism may be present on the same gene as an MPS VI mutation, acting as a second factor that may modify the disease progression of the pathologic mutation. Finally, there are a few documented occurrences where 2 or more disease-causing mutations are located on a single allele [62,63].

Historically Jin et al, (1992) [48] were the first to report mutations of the *ARSB *gene in rapidly progressing and slowly progressing phenotypes and suggested that genotype-phenotype correlations might be possible. Wicker et al, (1991) [18] described a report of a homozygous G137V mutation for an intermediate clinical phenotype with low but residual ASB enzyme activity, although no clinical description was given. Litjens et al, (1992) [64] reported a patient homozygous for an early frameshift mutation caused by the deletion of a G at position 238 (ΔG_238_) with a description of a rapidly progressing MPS VI phenotype as having a grossly elevated level of DS, deficiency of ASB in skin fibroblasts and peripheral blood leukocytes. The severe clinical features, which were noticeable within the first 2 days of age included dysmorphic facial features and dysostosis multiplex with abnormal vertebrae, small flared iliac wings, diphyseal distension of tubular bones and a large skull as the most prominent features. By 3 months of age the patient had corneal clouding, which worsened by 7 years of age leading to blindness. By 4 years of age the patient's upper airway obstruction was relieved somewhat by tonsillectomy and adenoidectomy. Also at 4 years of age the degree of hydrocephalus required shunting. At the age of 5 years joint contractures at the knees were relieved by surgery. By 6 years of age instability of the C2 and C3 vertebrae led to cervical cord compression, which required stabilization of these vertebrae. At age 11 years the patient underwent proximal femur extension osteotomies to relieve fixed flexion deformities of the hips bilaterally.

Despite limitations in genotype-phenotype correlations, progress has been made in developing technologies to assess or predict the impact of specific mutations on enzyme levels and activity. After molecular genetic analysis on DNA extracted from patients' skin fibroblasts using direct sequencing techniques to identify the *ARSB *mutations, these mutations were expressed in Chinese hamster ovary cells and analyzed for residual ASB activity and mutant ASB protein [15,65-68]. A second method has been developed to estimate the severity of missense mutations using 3-D visualization with Ras-Mol v2.7.3.1. software [69,70] to predict the effect of mutated amino acid substitutions on protein structure and function.

For nonsense mutations and deletions that disrupt protein production and cause enzyme deficiency the severity is clear. Missense mutations are harder to predict and need to be assessed directly with cell transfection and expression experiments or 3D modeling as described above to understand the impact of the mutation on the phenotype. Recently, *ARSB *DNA from a large MPS VI population representing approximately 10% of all known MPS VI patients has been sequenced. The study presents correlates of some genotypes with phenotype that have a much improved clinical phenotype description[15].

Despite some success making genotype-phenotype correlations for homozygous individuals, it is still not generally possible to predict phenotype from genotype[71]. For example, a case of two rapidly progressing mutations p.T92M and p.L498P in a compound heterozygote patient was described by Litjens et al, (1996) [72] that resulted in a slowly progressing phenotype. These alleles were subsequently expressed in a mammalian cell expression system, but the residual ASB activity and/or ASB protein representing the biochemical phenotype did not correspond to the observed clinical phenotype. Furthermore, the presence of two [62] or even three [63] disease-causing mutations in a single allele are reported and have been associated with rapidly progressing phenotype in MPS VI with concomitant high uGAG and low residual ASB activity. Recently a few correlations of phenotype to genotype, or correlation of elevated uGAG levels to genotype, have been described in the literature and are discussed below. Karageorgos et al 2007 [15] have identified the mutations for 105 patients participating in the cross-sectional survey of untreated MPS VI patients, and Phase 1, Phase 1/2 clinical trials. The Karageorgos study has identified 83 different disease-causing mutations, 62 of which were novel, with 11 deletions, 1 insertion, 7 splice site mutations, 4 polymorphisms, 5 nonsense and 38 missense mutations.

### Rapidly Progressing Genotype-Phenotype

The literature prior to the Karageorgos study revealed some well-studied homozygous missense mutations, deletions and insertions, and intronic mutations that had shown reasonable correlation to rapidly progressing phenotypes with severe symptoms: i.e., p.C117R [48]; ΔG238 [64]; p.G144R; p.C521Y; insertion T1285 [19]; and pL321P [62]. The Karageorgos study (2007) [15] provided additional mutations associated with severe phenotypes, with 3 homozygous forms; rapidly progressing clinical phenotypes were associated with missense mutations p.D54N, p.L98Q, and p.I223V. Also early onset, rapid progression, elevated uGAG with low ASB protein and activity levels were found in the same study to be associated with missense mutations p.L72R, and p.R315Q linked to the polymorphism p.S384N but caution should be taken when genotyping reveals p.S384N. Although some publications have reported this mutation to be associated with a rapidly progressing phenotype [15,63] there are no expression data available. Futhermore, Zanetti et al, 2009 recently identified p.S384N with significant frequency in a normal Italian population and have shown that p.S384N is a non-pathogenic polymorphism [73]. (This is also mentioned in the Unresolved Questions Section at end of document.)

### Slowly Progressing Genotype-Phenotype

It is difficult to relate genotype to phenotype for slowly progressing patients because progressive bone, joint and cardiac valve symptoms develop more slowly compared to the rate of symptom presentation for rapidly progressing patients. The Karageorgos study described the attenuated clinical phenotype as associated with patients carrying either the p.Y210C missense mutation or the p.C405Y missense mutation in independently heterozygous combinations [15]. These mutations were further associated with levels of uGAG below 100 μg/mg creatinine. This group of patients showed later onset and longer survival than rapidly progressing patients with levels of uGAG > 100 μg/mg creatinine [15]. No individuals homozygous for either p.Y210C or p.C405Y mutations have been reported; it is likely these individuals do not show signs of MPS disease and remain undetected in the general population. However, Karageorgos' study presented a group of patients older than 20 years of age with attenuated genotype, low uGAG, but poor endurance results for the 6-min walk test. These findings suggest that over time even slowly progressing patients may develop severe morbidity independent of their attenuated (slowly progressing) genotype. This is attributed to the accumulation of dermatan sulfate that contributes to progressive bone and joint disease, and cardiac valve disease. In particular, the bone and joint disease would impair performance in the walk test as a measure of endurance. For these reasons, treatment should not be based on genotype alone. Other mutations associated with uGAG levels below 100 μg/mg creatinine that were associated with attenuated disease progression were p.R152W[15] and C192R[19] when present in homozygous state; and p.D83Y[15], p.H430R[15] and p.R434I[15] when present in compound heterozygous states.

## Diagnosis, diagnostic methods, diagnostic criteria

Diagnosis requires the following:

• the analysis of ASB enzyme activity in isolated leukocytes or cultured skin fibroblasts at an accredited laboratory [61] to demonstrate large decrease or absence of ASB activity that is diagnostic for MPS VI. Although ASB enzyme activity levels may differ between testing laboratories, ASB activity in diagnosed MPS VI patients is generally less than 10% of the lower limit of normal ASB activity[29].

• the identification of normal enzyme activity of a different sulfatase. This excludes the diagnosis of multiple sulfatase deficiency (MSD).

Diagnosis is supported by the following:

• evidence of clinical phenotype: e.g. short stature, bone-related dysostosis multiplex, hepatosplenomegaly, macrocephaly, inguinal or umbilical hernia, corneal clouding or cardiac valve thickening. Clinical phenotype may not be evident in newborns or patients with a very mild phenotype[29]. It is important to note that a pseudo-deficient MPS VI gene mutation has been reported[52] with the patient demonstrating very low plasma ASB enzyme activity (exact level is not yet published), but normal uGAG and normal skeletal findings by X-ray supporting a normal phenotype.

• demonstration of elevated total uGAG at baseline that decreases significantly within 2 to 3 months of ERT administration, conducted at the same laboratory. UGAG are elevated in the newborn period and decrease over the first year, so this sign of response to ERT may not be useful in the newborn.

• DS accumulation and absence of CS, heparan sulfate, keratan sulfate or hyaluronate using thin layer chromatography (TLC), or high resolution electrophoresis fractionation.

• demonstration by an accredited laboratory of intermediate levels of leukocyte ASB enzyme activity in both parents to support diagnosis of carriers.

Confirmation by mutational analysis of the *ARSB *gene[15] should be considered if the diagnosis is in question, and is important in carrier testing or prenatal diagnosis.

## Differential diagnoses

Differential diagnoses within the MPS and mucolipidoses families of diseases include:

Mucopolysaccharidosis I H; I S; I H/S (Hurler syndrome; Scheie syndrome; Hurler-Scheie syndrome)

Mucopolysaccharidosis II (Hunter syndrome)

Mucopolysaccharidosis IVA (Morquio syndrome)

Mucopolysaccharidosis VII (Sly syndrome)

Multiple sulfatase deficiency (MSD)

Mucolipidosis I (now known as sialidosis), II, III, and IV

The mucopolysaccharidoses (MPS) I, II, IVA, VII and multiple sulfatase deficiency (MSD), may present early in life with similar symptoms as MPS VI. For diagnostic direction to aid in distinguishing MPS VI from other MPS see Table 2. MPS IVA may be distinguished from MPS VI by ligamentous laxity [74]. Composition of GAG substrates in urine may be helpful in differentiating the different MPS in combination with common differentiating physical features. MPS I patients typically have less severe corneal clouding than MPS VI [41]. MPS I may be differentiated from MPS VI by the excretion of both heparan sulfate and DS in urine in MPS I compared to near 100% DS in MPS VI. MPS II is an X-linked recessive disease affecting primarily boys, which is differentiated based upon the absence of corneal clouding and the presence of heparan sulfate and DS of equal amounts in urine. MSD may resemble MPS VI in early life but later the ichthyosis and mental deficits help differentiate MSD from MPS VI[75,76]. MSD usually have all GAG types present in urine since multiple sulfatase activities are simultaneously defective. To differentiate MPS VII, these patients are more often found to have clinical manifestations *in utero *or at birth, and corneal clouding is less common at ages below 8 years. MPS VII is also differentiated based on uGAG comprised of heparan sulfate, chondroitin 4-, and 6-sulfates in addition to DS. Mucolipidosis I (better known as sialidosis), III and IV are differentiated from MPS VI by absence of increased GAG in urine. ML II may have increased uGAG with high levels of beta-hexosaminidase, iduronate sulfatase and arylsulfatase A at 10-20 times the reference range in serum but deficiency of the same enzymes in cultured fibroblasts. [Note: the relevant amount of the detected GAG DS, heparan sulfate and CS, is often dependent on the method used to assess uGAG and also the patient's age].

**Table 2:** Differential Diagnosis of MPS VI.

Disease	Typical Age at Diagnosis	Typical Life Expectancy	Inheritance	Common Differentiating Physical Features	Intelligence and Behaviour	Excess GAG excreted in urine
MPS I Hurler (IH), Scheie (IS), and Hurler-Scheie (IH-IS) syndrome	▪IH: Infancy (before 1 year old) ▪IS: between 10 and 20 years of age ▪IH-IS: between 3 and 8 years of age	▪IH: Death in childhood. ▪IS: Normal lifespan. ▪IH/IS: Death in early adulthood	▪Autosomal recessive	▪Stature ranges from extremely short after 1 year of age to short ▪Skeletal abnormalities range from severe in IH to stiff joints in IS ▪Very coarse to moderately coarse facial features ▪Corneal opacities	▪IH: Severe mental retardation ▪IS: Normal intelligence ▪IH/IS: Normal intelligence	▪Heparan sulfate and <70% dermatan sulfate

MPS II (Hunter Syndrome)	▪Severe disease: 1 to 2 years of age	▪Severe disease: death before 15 years of age ▪Attenuated disease: survival into adulthood	▪X-linked recessive	▪Stature ranges from moderately short stature after 1 year of age to short ▪Marked skeletal abnormalities in severe disease ▪Coarse facial features ▪Absence of corneal opacities ▪Pebbly ivory skin lesions in some patients	▪Severe disease: marked mental retardation after 1 year of age ▪Attenuated disease: normal intelligence	▪Heparan sulfate and <50% dermatan sulfate

MPS III (Sanfilippo A, B, C, and D syndrome)	▪4 to 6 years of age	▪Death in puberty is common	▪Autosomal recessive	▪Normal stature ▪Mild skeletal abnormalities ▪Mild coarseness of facial features ▪Absence of corneal opacities	▪Profound mental deterioration, especially after 3 years of age ▪Hyperactivity	▪Heparan sulfate

MPS IV (Morquio syndrome A and B)	▪1 to 3 years of age	▪Survival ranges from childhood to middle age	▪Autosomal recessive	▪Extreme short stature after 1 year of age ▪Skeletal abnormalities are distinctive ▪Hypoplasia of tooth enamel ▪Mid-face hypoplasia and mandibular protrusion ▪Corneal opacities	▪Normal	(A) Keratan sulfate and chondroitin 6-sulfate (B) Keratan sulfate

MPS VII (Sly syndrome)	▪Neonatal to childhood	▪Survival ranges from infancy to at least the fourth decade of life	▪Autosomal recessive	▪Skeletal abnormalities ▪Hepatosplenomegaly ▪Hydrops fetalis is a common form of presentation	▪Intellectual deficits	▪Dermatan sulfate ▪Heparan sulphate ▪Chondroitin 4-, 6-sulfates

MPS IX	▪Adolescence	▪Not known, as only 1 adolescent patient identified in literature	▪Autosomal recessive	▪Short stature ▪Periarticular masses in soft tissue	▪Normal	▪Hyaluronan

Multiple Sulfatase Deficiency (also called mucosulfatidosis or Austin syndrome)	▪By 2 years for severe diseases	▪Death during the first decade of life	▪Autosomal recessive	▪Skeletal abnormalities usually are severe ▪Hepatosplenomegaly ▪Loss of retinal pigment, grey maculae, and optic atrophy ▪Neurodegenerative signs with demyelination lead to a vegetative state and death ▪Ichthyosis	▪Neuro-degenerative disease leads to a vegetative state	▪Glycosaminoglycans (GAG)

[21]

## Prenatal Diagnosis, Newborn Screening and Genetic Counseling

Prenatal diagnosis is based primarily on reduced/low ASB activity with optional support of known mutational analysis for at-risk fetuses where a family already has a child with MPS VI. Affected members of such a family usually have the same genotype. Diagnostic testing may be conducted for prenatal diagnosis on viable fetal cells from chorionic villi, cultured amniotic fluid cells or in the newborn on a dried blood spot[77] sampled via the heel stick method or as recently described, via cordocentesis [R Giugliani, personal communication], and is available for MPS VI at a few select laboratories around the world[78]. Prenatal studies are limited to families having a previous child with MPS VI.

With the availability of specific FDA-approved therapies for lysosomal storage diseases (Gaucher disease [imiglucerase, Cerezyme^® ^U.S. Prescribing Information, Genzyme Corp., 1994], Fabry disease [agalsidase beta, Fabrazyme^® ^U.S. Prescribing Information, Genzyme Corp., 2003], Pompe disease [alglucosidase alfa, Myozyme^® ^U.S. Prescribing Information, Genzyme Corp., 2006], MPS I [laronidase, Aldurazyme^® ^U.S. Prescribing Information, BioMarin Pharmaceutical Inc./Genzyme Corp., 2003], and MPS VI [galsulfase, Naglazyme^® ^U.S. Prescribing Information, BioMarin Pharmaceutical Inc., 2005][79]), there is much greater need to have accurate, early diagnoses. Furthermore, recent studies of galsulfase administration in MPS VI animal models from birth[55,80] and in neonatal MPS VI patients from as young as approximately 8 weeks of age [81] have indicated that very early initiation of ERT leads to better long-term outcomes[68,8].

Newborn screening is in pilot testing in several collaborating research laboratories in the US, Europe and Australia. It is based upon two primary methodologies: enzyme protein immunoassay based on microbead array technology or enzyme activity assay detected by tandem mass spectrometry. Screening assays have already been described using dried blood spots for other lysosomal storage disorders with available treatments i.e., MPS II [82], MPS I, Gaucher, Krabbe, Niemann-Pick A/B, Fabry, and Pompe diseases [83-86].

## Management

### Supportive care and surgical intervention

Until recently, supportive care and bone marrow transplantation were the only therapies available for MPS VI patients. Supportive care has focused on optimizing general health with nutrition counseling, occupational and physical therapy, and management of individual symptom complications, such as respiratory insufficiency requiring oxygen and/or positive airway pressure during sleep, tonsillectomy, adenoidectomy or tracheostomy, cardiac failure requiring medication and/or valve replacement if indicated, spinal cord compression or carpal tunnel compression requiring surgical decompression, corneal clouding requiring transplantation, or hydrocephalus requiring insertion of ventriculoperitoneal shunt. Additional disease management information is contained in this manuscript in the Clinical Description Section as well as in more detail in the Management Guidelines for Mucopolysaccharidosis VI by Giugliani et al, (2007) [26] and available online [87].

### Bone marrow transplantation

Bone marrow or hematopoietic stem cell transplantation (HSCT) has been used in rare cases in the past 24 years to treat MPS VI [88-95] with one long-term follow-up reported in four MPS VI patients[96]. Although leukocyte ASB enzyme level and uGAG improve, skeletal abnormalities remain difficult to stabilize or correct. Bone marrow transplantation (or HSCT) has been limited by the risks of death, morbidity from chronic graft-versus-host disease and difficulty obtaining adequate or optimal HLA-matched donors [97]. Between 1982 and 2007, there have been 45 MPS VI patients from around the world, (U.S., Saudi Arabia, Brazil, England, China, Australia, and Japan), registered with the Center for International Blood and Marrow Transplant Research (CIBMTR), and who received allogeneic stem cell transplantation. The 1-year survival for these patients was 67% (95% CI: 53-80) (CIBMTR, unpublished data).

It will be important in the future to compare long-term outcomes of MPS VI patients treated with HSCT with patients treated at an early age with ERT. With the introduction of ERT, two rapidly progressing MPS VI patients who had previously received HSCT, but who had experienced failure of engraftment were transitioned to ERT, with no adverse events related to ERT during follow-up to 36 and 80 weeks, respectively [98].

### Enzyme replacement therapy

ERT using genetically engineered human enzyme manufactured by recombinant DNA technology in high-output cell lines is an established treatment for several lysosomal storage diseases based on the unique ability of human cells to bind and transport exogenous enzyme into the lysosomal compartment. In the case of MPS VI, human ASB is produced and marketed as the drug Naglazyme^® ^(galsulfase); it utilizes the mannose-6-phosphate receptor to bind at the cell surface and traffic intracellularly to lysosomes, and effectively replaces the absent or deficient ASB enzyme. Although galsulfase is able to reach most tissues after IV infusion, the CNS, cornea and articular cartilage are excluded by the blood-brain barrier or poor vascularization. Extensive preclinical studies were completed in a feline model of MPS VI [80] that established the dose, safety, as well as efficacy in reversing storage, and limiting bone disease when started very early in life. These preclinical studies were followed by Phase 1/2, Phase 2 and Phase 3 human clinical trials [99-102]. Significant improvements in 12-minute-walk test, nearly significant improvement in 3-minute stair climb and significant reduction in uGAG levels were demonstrated in the 24-week, randomized, double-blind Phase 3 trial [102]. These studies led to approval of Naglazyme^® ^in the US (May, 2005), the EU (January, 2006), Australia (March, 2007), Croatia (June, 2007), Switzerland (October, 2007), South Korea (January, 2008), Japan (March, 2008), Belarus (August, 2008), Brazil (February, 2009), Algeria (June, 2009), and Russia (August, 2009). As of September, 2009, Naglazyme^® ^has been marketed in 39 countries.

On long-term follow-up, improvement in endurance on the walk test and stair climb, and reduction in uGAG[103], positive effects on puberty and growth (Decker C et al, unpublished data), and pulmonary function [104] were maintained in clinical study subjects from all trials, with observation times up to a maximum of 5 years with regular Naglazyme^® ^therapy. A case study has shown improvement following ERT of pulmonary function leading to a reversal of tracheostomy intubation in an MPS VI patient [24]. Another case study has shown reversal of papilledema and improved vision in an 11-year old MPS VI patient followed on ERT maintained during follow-up of 130 weeks [105]. A case series of 6 MPS VI patients showed stabilization of visual acuity after treatment with ERT for 144 weeks [106].

ERT with Naglazyme^® ^is generally considered to have an acceptable safety profile[79]. As is commonly observed for this class of intravenous protein therapeutics, infusion reactions have occurred during administration of Naglazyme^®^, but are generally mild to moderate in severity, and can be managed by stopping or slowing infusion, and administering some combination of pretreatment with antihistamines, steroids and/or antipyretic agents [107]. More detailed information regarding treatment may be found in the recently published Management Guidelines for Mucopolysaccharidosis VI by Giugliani et al, 2007 [26] available on the Internet [87] and in galsulfase, Naglazyme^® ^U.S. Prescribing Information, BioMarin Pharmaceutical Inc., 2005[79].

In order to examine the effect of very early ERT on the skeletal dysplasia and other manifestations of the disease, a sibling pair with MPS VI was followed. The newborn infant, who was identified early due to the knowledge that the first child had been diagnosed with MPS VI, was started on ERT administered within the first two months of life [81]. This MPS VI neonate treated from almost 8 weeks of age for 3.6 years showed lack of scoliosis, normal joint range of motion, and normal cardiac valves as compared to the older sibling started on ERT at 3.6 years of age and treated with ERT for 3.6 years. The observations in this sibling study underscore the critical need for early diagnosis and intervention, which in most cases will require newborn screening. Further support for early administration of ERT may be forthcoming from a recent study of infants started on Naglazyme^® ^(galsulfase) at ages below 1 year (C Decker, personal communication).

Administration of ERT Naglazyme^® ^is generally performed in a medical setting because of risk of allergic reaction [107] and the need to have specialized services to place intravenous access for weekly infusion. Furthermore, care should be taken when considering administration of prophylactic antihistamines for the prevention of infusion-associated reactions to ERT since diphenhydramine may result in serious sleep apnea (USPI). Recently, patients with less severe symptoms and absent infusion-related reactions have been treated in the home setting [108-110]. Although this treatment option may provide improved convenience for some patients, it is clearly a decision that patient, parent(s) or guardian(s) and treating physician will need to make together, recognizing the risks of possible allergic reaction and possible need for placement of permanent central venous access (i.e., mediport). Central venous access devices have risks of infection and venous thrombosis. Families should be aware of these risks and seek medical evaluation with any unexplained fever, or swelling of face or extremity.

## Prognosis

Prognosis probably depends upon age at onset of first symptoms, rate of disease progression, age at start of treatment with ERT or HSCT, and experience of medical and surgical staff caring for the patient. In both rapidly and slowly progressing forms patients may suffer irreversible damage if disease goes undetected and undiagnosed, and if ERT initiation is consequently delayed. In the large Swiedler Survey study of 121 untreated patients there were fewer patients aged over 20 years, but those few over 20 years of age were found to have uGAG below 100 μg/mg creatinine; whereas there were substantially more patients in their teenage years and younger with uGAG above 100 μg/mg creatinine[14]. Thus, uGAG above 100 μg/mg creatinine is considered to be a negative prognostic sign. Slowly progressing patients may generally survive until the third or later decades. Very long-term studies documenting the effect of ERT on age of death have not yet been completed although results of long-term follow-up while on ERT with respect to endurance-related walk tests and uGAG (92 to 260 weeks)[103], puberty and growth (240 weeks) (Decker C et al, unpublished data) and pulmonary function (240 weeks) [104] have been submitted, recently accepted or published by journals at the time of writing. The Clinical Surveillance Program (CSP) was established by BioMarin Pharmaceutical Inc. in 2005 to study the natural history of MPS VI in Naglazyme^®^-treated and untreated patients in the US and EU for a period post-launch of 15 years. Observations made in this program may provide insight into the question about the long-term effect of therapy in the coming years.

## Unresolved questions

Correlation of genotype to phenotype and prediction of severity have limitations due to the low frequency of homozygosity, high frequency of compound heterozygosity, incomplete identification of polymorphisms, and the occasional finding of multiple mutations on a single allele. This is an active area of research and for these reasons the literature is likely to be quickly outdated and updated frequently on these topics. For example, some mutations previously associated with rapidly progressing MPS VI have recently been shown to be common polymorphisms. The 1151G > A (p.S384N) mutation was most often linked to the pathogenic p.R315Q on the same allele and has been described in MPS VI patients and family members at risk [70,73]. However, it is important to note that Garrido et al, 2008 [70] and Zanetti et al, 2009 [73] have subsequently shown that p.S384N is not a mutation but a non-pathogenic polymorphism. Additionally, p.S384N appears as a polymorphic variant in the Central European panel of the HapMap (haplotype mapping) project [111]. This new finding that p.S384N is non pathogenic has implications for the future development of the anticipated newborn screening, and for existing prenatal diagnosis (in the case of a family with a known MPS VI child) that may have led in the past to incorrect correlations of genotype to phenotype regarding these polymorphisms and mutations. The heterogeneity of MPS VI and the current inability to consistently and reliably link genotype to phenotype for compound heterozygotes, mean that it is inadvisable to define appropriate access to specific therapies based on genotype at this time.

Although ERT with Naglazyme^® ^has been successful in treating deficits of endurance [103], pulmonary function [104], growth and puberty (Decker C et al, unpublished data), it has not been able to resolve the symptoms of MPS VI disease occurring in certain regions of the CNS, ophthalmological system and joints due to the limitations of the blood-brain barrier and comparatively poor vascularization of the joints preventing penetration of enzyme to these regions. Recent studies that challenge these limitations may lead to future successes. For example there are recent publications and communications at MPS conferences that report on long-term intra-articular ERT in MPS VI cats [112,113], combined intrathecal ERT with intravenous (IV) ERT in MPS VI cats (Auclair D et al, unpublished data); intrathecal delivery of ERT to the brain in MPS I dogs [114]; and combined intrathecal ERT with intravenous (IV) ERT in MPS I dogs from birth (Dierenfeld A et al, unpublished data). Furthermore there has been one case study of intrathecal administration of ERT to an MPS I patient [51] and a case study of intrathecal administration of ERT to an MPS VI patient [115]. All these reports have shown promising results. Human clinical trials examining these therapeutic approaches are anticipated but are not yet initiated. MPS animal model studies from birth in MPS VI cats[55], MPS I dogs (Dickson P et al, unpublished data), and a single case study of a human patient with rapidly progressing form of MPS VI from the first months of life [81] have shown limited evidence that very early intravenous treatment of MPS VI disease with ERT can reduce bone disease and improve skeletal development. Larger trials requiring identification of patients through newborn screening will be critical [116] to prove effectiveness of treatment in preventing the skeletal dysplasia that is the primary manifestation of MPS VI disease.

## Abbreviations

MPS: mucopolysaccharidosis/mucopolysaccharidoses; GAG: glycosaminoglycan(s); uGAG: urinary glycosaminoglycan(s); ASB: arylsulfatase B; *ARSB*: arylsulfatase B (gene/mutation); DS: dermatan sulfate; CS: chondroitin 4-sulfate; ERT: enzyme replacement therapy; MSD: multiple sulfatase deficiency; LSD: lysosomal storage disorder; CNS: central nervous system; GM: ganglioside; ML: mucolipidosis; CSP: clinical surveillance program; MRI: magnetic resonance imaging; ICP: intracranial pressure; IOP: intraocular pressure; HSCT: hematopoietic stem cell transplantation, HLA: human leukocyte antigen; HapMap: haplotype mapping; indel insertion and deletion; ENT: ear nose and throat; CIBMTR: Center for International Blood and Marrow Transplant Research.

## Competing interests

VV and PH received honoraria for presentations given on behalf of BioMarin Pharmaceutical Inc. PH was a principal investigator on 3 galsulfase clinical trials and has provided consulting support to BioMarin Pharmaceutical Inc. PH receives support, in part, from NIH/NCRR UCSF-CTSI Grant Number UL1 RR024131. The manuscript's contents are solely the responsibility of the authors and do not necessarily represent the official views of the NIH.

HN and ST are employees and stockholders of BioMarin Pharmaceutical Inc.

## Authors' contributions

VV and PH added substantial intellectual, clinical information and reviewed the document for accuracy. HN is a medical writer, who conducted a literature search, compiled and incorporated references into the Reference section, prepared and wrote substantial pieces of the document, and edited the final document for review and submission. ST contributed to conception and design, acquired data, and critically reviewed the document from the beginning to the end of preparation.

All authors have approved the final manuscript.

## Consent

Written consent for publications of photographs was obtained from the patients or legal guardians where required.
